# Pressure-Dependent Mechanical Behavior and Surface Degradation of Fluorocarbon Elastomer (FKM): Insights into Structure–Property Relationships Under Hydrogen Exposure

**DOI:** 10.3390/polym18101253

**Published:** 2026-05-21

**Authors:** Nitesh Subedi, Alfredo Becerril Corral, Md Monjur Hossain Bhuiyan, Md Ariful Islam, Zahed Siddique

**Affiliations:** School of Aerospace and Mechanical Engineering, University of Oklahoma, Norman, OK 73019, USA

**Keywords:** hydrogen exposure, FKM elastomer, polymer degradation, mechanical behavior, surface morphology, structure–property relationship

## Abstract

This study investigates the pressure-dependent mechanical behavior and surface degradation of fluorocarbon elastomer (FKM, Viton^®^) O-ring seals following prolonged high-pressure hydrogen exposure. Specimens were aged at up to 7000 psi for 192 h and evaluated using tensile testing and optical image analysis. The results show a non-monotonic evolution of peak force, stiffness, and energy absorption, with increased load-bearing response at higher pressures accompanied by reduced displacement capacity. Normalized force–displacement behavior shows broadly similar loading profiles across pressure conditions; however, this representation is used for comparative visualization and does not establish preservation of the deformation mechanism. Image-based analysis reveals a significant increase in micro-defect density and surface heterogeneity with pressure, suggesting increased formation of surface micro-defects. These findings highlight pressure-dependent changes in polymer network response and surface morphology under hydrogen exposure. The study provides insights into structure–property relationships governing elastomer performance in hydrogen environments.

## 1. Introduction

### 1.1. Background and Motivation

Hydrogen is increasingly recognized as a critical energy carrier for enabling the transition toward low-carbon energy systems across transportation, power generation, and industrial sectors [1,2,3]. Due to its low ambient density, practical storage and transport require compression to high pressures, introducing significant challenges related to material reliability and long-term system integrity. Under such conditions, elastomeric sealing components such as O-rings and gaskets are widely used in valves, connectors, and storage assemblies to prevent gas leakage and ensure operational safety [4,5].

Elastomeric materials provide effective sealing through their ability to deform under compression and conform to mating surfaces [6]. However, exposure to high-pressure hydrogen environments can alter their mechanical and physical behavior. These changes may include softening, swelling, loss of stiffness and, in severe cases, cracking or rupture during decompression [7,8,9]. Such degradation mechanisms can compromise sealing performance and increase the risk of leakage.

Fluorocarbon elastomers (FKM, Viton^®^) are widely used in demanding sealing applications due to their resistance to aggressive chemicals, elevated temperatures, and relatively low gas permeability [6]. The fluorinated polymer backbone contributes to chemical stability and reduced transport of gases compared to many non-fluorinated elastomers. Nevertheless, hydrogen can still diffuse into the polymer network under high-pressure conditions, leading to physical changes such as swelling and temporary plasticization, which may influence mechanical response and sealing performance [7,9].

The mechanical behavior of elastomeric seals plays a critical role in maintaining sealing integrity. The contact pressure at the seal–metal interface depends directly on the elastic modulus and load-bearing capability of the material. Any reduction in stiffness or tensile resistance can reduce sealing force and increase the likelihood of leakage. In addition, under cyclic loading or rapid decompression, mechanically degraded seals may undergo failure modes such as extrusion, blistering, or rupture [4,8].

In practical service environments, elastomeric sealing materials are rarely subjected to monotonic loading; instead, they experience repeated loading–unloading cycles due to dynamic pressure, vibration, and mechanical actuation. Such cyclic deformation induces nonlinear mechanical responses, including stress softening (Mullins effect), hysteresis, and progressive structural evolution within the polymer matrix and filler network [10,11]. These processes alter chain orientation, filler–polymer interactions, and internal microstructure, ultimately influencing macroscopic mechanical behavior and durability. Accordingly, consideration of cyclic deformation is essential for representing realistic service conditions and assessing long-term material performance.

Under hydrogen exposure, these mechanical responses are further influenced by coupled transport–mechanical phenomena, including gas sorption, diffusion, and volumetric swelling. These processes introduce time-dependent changes in stiffness, strength, and displacement behavior [7,9,12,13]. Consequently, the combined effects of cyclic deformation and hydrogen-induced transport mechanisms govern the pressure-dependent mechanical response of elastomeric sealing materials, making their systematic evaluation critical for ensuring reliable performance in hydrogen systems. This study therefore investigates the pressure-dependent mechanical behavior and surface degradation of fluorocarbon elastomer O-rings, with emphasis on linking macroscopic response to underlying microstructural evolution.

### 1.2. Contemporary Research on Hydrogen–Elastomer Degradation

Recent research has shown that degradation of elastomers in high-pressure hydrogen environments is strongly influenced by coupled transport–mechanical interactions, particularly during pressure cycling and decompression [4,7,8,9]. While hydrogen uptake during pressurization may not immediately lead to failure, rapid depressurization can generate localized internal stresses that result in irreversible damage such as microvoid formation, blistering, and internal cracking [8,9,13]. The severity of this damage is governed by factors including pressure history, decompression rate, temperature, and exposure duration.

A key aspect emphasized in the literature is the distinction between reversible and irreversible material responses. Mechanical changes associated with transient hydrogen sorption and plasticization may partially recover after degassing, whereas permanent damage-such as surface defects, microstructural alterations, or increased compression set-indicates irreversible degradation [7,9,14]. This distinction is essential for interpreting post-exposure mechanical measurements and evaluating long-term sealing reliability.

Despite advances in understanding hydrogen-induced damage mechanisms, systematic evaluation of post-exposure mechanical behavior remains limited. In particular, tensile response of elastomeric sealing materials under realistic conditions is not widely reported [4,7]. Furthermore, many studies rely on simplified specimen geometries that do not fully capture the behavior of actual sealing components. This gap highlights the need for experimental investigation using complete O-ring geometries to better represent practical sealing applications.

### 1.3. Knowledge Gap

Despite advances in understanding hydrogen-induced degradation mechanisms, direct evaluation of post-exposure mechanical performance of elastomeric sealing materials remains limited. In particular, systematic investigation of how high-pressure hydrogen exposure affects uniaxial tensile response is not widely reported. This gap is critical because tensile behavior directly governs load-bearing capability and sealing integrity under service conditions.

Furthermore, most existing studies are conducted using simplified specimen geometries, which do not fully represent the behavior of actual sealing components. As a result, the applicability of these findings to real engineering systems remains uncertain. There is therefore a need for controlled experimental studies that directly evaluate pressure-dependent mechanical response using realistic sealing geometries.

### 1.4. Objectives of This Study

The objective of this study is to systematically evaluate the mechanical response of fluorocarbon elastomer (FKM, Viton^®^) sealing materials following controlled high-pressure hydrogen exposure. The focus is on quantifying changes in uniaxial tensile behavior, including force–displacement response, load-bearing capacity, and deformation characteristics.

To achieve this, experiments are conducted across multiple hydrogen pressure levels under consistent exposure conditions, enabling direct assessment of pressure-dependent trends in material behavior. The maximum exposure pressure considered is 7000 psi (≈48 MPa), representing conditions relevant to hydrogen compression, storage, and distribution systems.

Unlike conventional approaches that rely on standard test specimens, this study evaluates complete O-ring geometries to better represent practical sealing components. By directly measuring mechanical response in realistic configurations, this work aims to provide experimentally grounded insight into hydrogen-induced changes in FKM elastomers and to support improved design and reliability of sealing systems.

## 2. Materials and Methods

The materials used in this study and the experimental procedures for hydrogen exposure and post-aging characterization are described in the following sections.

### 2.1. Materials

Commercial fluorocarbon elastomer (FKM, Viton^®^) O-rings were used as the sealing material in this study. The O-rings were procured from McMaster-Carr (Elmhurst, IL, USA) (part number 1284N17) and conform to SAE AS568 dimensional standards (Dash-214). The material corresponds to a commercially available soft-grade Viton^®^ fluoroelastomer (FKM, 60 Shore A), commonly used in industrial sealing applications and designed for improved compressibility and sealing performance under moderate pressure conditions.

The FKM O-rings correspond to the AS568 Dash-214 size and have an inner diameter of 0.984 in (25.0 mm), an outer diameter of 1.262 in (32.05 mm), and a cross-sectional diameter of 0.139 in (3.53 mm). The use of standardized O-ring geometry ensures consistency with industrial sealing applications and enables direct evaluation of mechanical behavior in realistic component configurations.

Detailed information regarding batch-specific formulation, molecular weight, and processing conditions was not available from the supplier, which is a common limitation when using commercially sourced elastomeric materials.

The key physical and specification parameters of the FKM O-ring specimens used in this study are summarized in Table 1.

**Figure 1:**
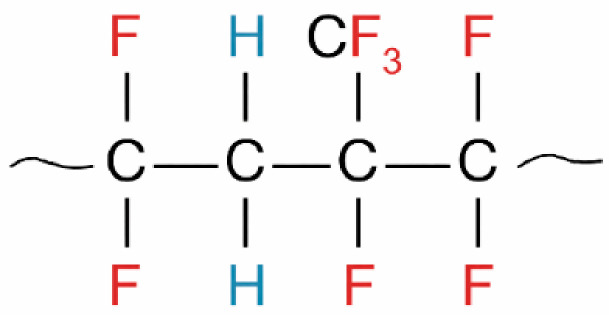
Representative chemical structure of fluorocarbon elastomer (FKM (Viton^®^) showing the fluorinated polymer backbone composed of vinylidene fluoride (VDF) and hexafluoropropylene (HFP) units. The high density of carbon–fluorine (C–F) bonds contributes to the material’s chemical resistance, thermal stability, and reduced gas permeability.

Fluorocarbon elastomers (FKM), commonly referred to as Viton^®^, are high-performance elastomers composed primarily of copolymers based on vinylidene fluoride (VDF) and hexafluoropropylene (HFP). As illustrated in Figure 1, the polymer backbone contains a high density of carbon–fluorine (C–F) bonds, which provide excellent resistance to chemical attack, thermal degradation, and oxidative environments. The presence of bulky fluorine atoms results in a relatively dense molecular structure with reduced chain mobility and lower free volume compared to many non-fluorinated elastomers, contributing to lower gas permeability and improved environmental stability.

Despite these advantages, small gas molecules such as hydrogen can diffuse into the polymer network under high-pressure conditions, leading to physical changes such as swelling, temporary plasticization, and stress development during depressurization. These effects can influence both mechanical response and surface integrity.

Commercial FKM compounds typically incorporate reinforcing fillers, curing agents, and stabilizers to enhance mechanical performance and durability. The resulting crosslinked polymer network provides the elastic recovery required for sealing applications under load. Because the present study investigates commercially manufactured O-rings with standardized geometry and industrial specifications, the measured responses are representative of practical sealing materials used in real applications.

The relatively high fluorine content and moderate hardness of the selected FKM grade are expected to influence both hydrogen transport behavior and pressure-dependent mechanical response, which are examined in the subsequent sections.

### 2.2. Rationale for Material Selection

Fluorocarbon elastomer (FKM, Viton^®^) was selected in this study due to its widespread use in high-performance sealing applications involving aggressive environments, elevated temperatures, and high-pressure gases. In hydrogen infrastructure systems, elastomeric seals are frequently subjected to such conditions, making material selection critical for ensuring long-term sealing integrity and safety.

FKM is commonly employed in these applications because of its combination of chemical stability, mechanical robustness, and relatively low gas permeability compared to many conventional elastomers. As a result, it serves as a representative industrial sealing material for evaluating hydrogen–elastomer interactions. The selection of FKM in this study is therefore motivated by the need to investigate the behavior of a widely used sealing material under controlled hydrogen exposure conditions. By focusing on a single material system with consistent geometry and properties, the present work isolates the intrinsic effect of hydrogen pressure on mechanical response, providing a clear baseline for assessing performance in practical applications. The key specimen characteristics, hydrogen aging conditions, and tensile testing parameters used in this study are summarized in Table 2.

### 2.3. Hydrogen Aging Protocol

Hydrogen aging experiments were conducted using a sealed high-pressure stainless-steel chamber designed for controlled gas exposure of elastomer specimens. High-purity hydrogen gas was supplied from a compressed gas cylinder and pressurized to the desired levels using a high-pressure gas booster. Aging pressures were systematically varied from 800 psi to 7000 psi to investigate pressure-dependent effects on material behavior.

The exposure system was equipped with a dedicated inlet port for hydrogen pressurization and a separate port for thermocouple insertion to continuously monitor internal temperature. A vent line positioned on the opposite side of the chamber enabled controlled depressurization following exposure. For safety, the setup incorporated a hydrogen detection system integrated with an automatic shut-off valve, which isolated the chamber in the event of hydrogen leakage. All experiments were conducted at room temperature for a constant duration of 192 h, with no external heating applied, in order to isolate pressure-driven aging effects under isothermal conditions.

The experiments were performed using a high-pressure laboratory reactor (model GC-9, High Pressure Equipment Company, Erie, PA, USA). The reactor is a confined gasket-closure pressure vessel constructed from 316 stainless steel with an internal volume of approximately 500 mL. It has an internal diameter of approximately 2 in. and a depth of approximately 10 in, allowing multiple O-ring specimens to be exposed simultaneously under uniform gas conditions. The vessel is rated for pressures exceeding 12,000 psi at ambient temperature, ensuring safe operation within the pressure range used in this study.

**Figure 2:**
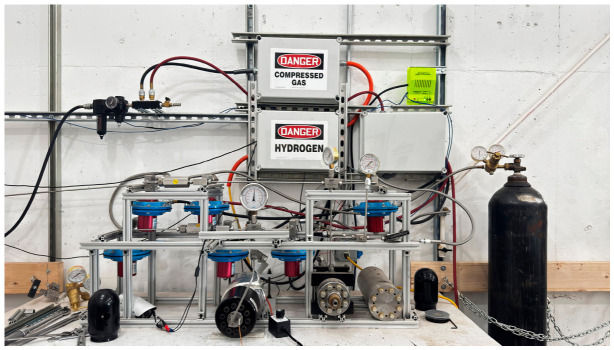
High-pressure hydrogen aging system used for elastomer exposure. Experimental setup including the compressed hydrogen supply, pressure regulation components, safety system, and exposure assembly used to age elastomer O-ring specimens under controlled conditions. The high-pressure reactor is the black cylindrical vessel mounted horizontally at the base of the setup, which serves as the sealed pressure chamber for hydrogen aging. It is connected to the gas supply through the piping system, while pressure gauges, valves, and associated fittings enable monitoring and regulation of internal pressure during testing.

The overall hydrogen exposure system is shown in Figure 2, with the reactor chamber located centrally beneath the pressure gauge. The vessel consists of a thick-walled stainless-steel body sealed by a bolted cover assembly using a metal gasket and thrust ring, enabling leak-tight containment of hydrogen at elevated pressures while maintaining uniform exposure conditions for the elastomer specimens.

During the exposure period, the chamber was maintained at constant pressure without intermediate pressurization–depressurization cycles. At the end of the 192 h aging period, the system was depressurized in a controlled manner through the vent line. Depressurization was achieved by opening the vent valve and allowing gas to escape through a restricted outlet orifice, resulting in an approximate pressure decay rate of ~70 bar min^−1^ until atmospheric pressure was reached.

This depressurization rate is higher than those typically used in standardized rapid gas decompression (RGD) qualification tests, which generally employ controlled rates on the order of 20–40 bar min^−1^ under cyclic loading conditions. In the present study, the objective was not to replicate standardized RGD testing protocols but to evaluate post-exposure mechanical behavior following prolonged hydrogen saturation. The relatively rapid pressure release represents a conservative condition that may enhance transient gas-related effects such as internal pressurization and plasticization. Because identical exposure and depressurization conditions were applied to all specimens, the resulting trends enable consistent evaluation of pressure-dependent mechanical response in FKM (Viton^®^) sealing materials.

Following depressurization, specimens were removed from the chamber and mechanically tested under ambient laboratory conditions within a short time interval to minimize uncontrolled hydrogen desorption. The elapsed time between chamber depressurization and tensile testing was approximately 5–10 min, corresponding to the minimum handling time required for specimen transfer and installation. During this interval, hydrogen desorption may initiate but remains limited due to the relatively slow diffusion kinetics in crosslinked elastomer networks.

Because mechanical testing was conducted after removal from the pressure vessel, specimens were not maintained under hydrogen pressure during testing. However, residual dissolved hydrogen is expected to remain within the elastomer immediately after decompression. Consequently, the measured mechanical response reflects a near-saturated post-exposure condition and may include contributions from hydrogen sorption, transient plasticization, and potential microstructural changes induced during high-pressure exposure.

The selected pressure range (800–7000 psi) provides a systematic multi-level dataset while maintaining constant exposure duration, temperature, and testing conditions. The upper pressure limit corresponds to the maximum stable operating condition for long-duration experiments in the present system and enables evaluation of pressure-dependent mechanical behavior under conditions relevant to hydrogen compression and storage applications.

It should be noted that an unexposed (0 psi) control condition was not included in this study. The experimental design focused on evaluating pressure-dependent trends under hydrogen exposure, with 800 psi serving as the lowest baseline condition. Consequently, the reported responses are interpreted relative to this baseline rather than an unaged reference state.

### 2.4. Tensile Testing of O-Ring Specimens

Mechanical characterization of the elastomer specimens was conducted using uniaxial tensile testing performed directly on complete O-ring geometries. Instead of preparing conventional dumbbell specimens, the testing approach utilizes the actual sealing configuration, enabling evaluation of mechanical behavior under conditions representative of practical applications. In this study, cyclic loading refers specifically to mechanical cyclic tensile loading of the O-ring specimens under displacement-controlled conditions, rather than pressure cycling within the hydrogen chamber.

The testing methodology is based on the principles outlined in ASTM D1414 [15], which describes the determination of tensile properties of elastomeric O-rings using a spool-type fixture. In this configuration, the O-ring is mounted over two cylindrical spools attached to the grips of a universal testing machine. This arrangement allows the specimen to undergo tensile deformation in the circumferential direction while reducing localized stress concentrations that would otherwise arise from direct clamping.

Prior to testing, the distance between the spools was adjusted to allow installation of the O-ring without introducing initial strain. Tensile loading was then applied under displacement-controlled conditions at a constant deformation rate of 0.508 mm/s, consistent with standard testing practice. The term “displacement” refers to the crosshead movement recorded during testing and is used consistently throughout this study. As the test progresses, the spools are displaced apart, generating a uniform tensile load along the ring circumference.

The effective deformation of the specimen is defined based on the changing center-to-center distance between the spools during loading. Following standard O-ring testing methodology, the effective gauge length is expressed as the sum of twice the spool spacing and the circumference of a single spool. Force–displacement data were continuously recorded, and the mechanical response of the material was evaluated using the measured peak force and corresponding displacement.

**Figure 3:**
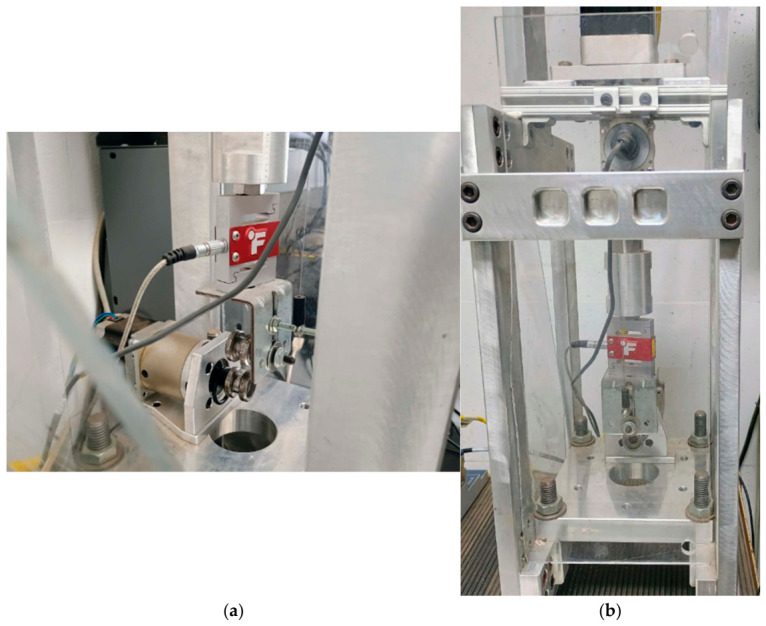
Tensile testing configuration for O-ring specimens. Experimental setup used for uniaxial tensile testing of elastomer O-rings using cylindrical spool grips, enabling circumferential loading while minimizing stress concentration at contact regions. (**a**) Side-view image of the tensile testing configuration showing the cylindrical spool grip arrangement used for O-ring loading. (**b**) Front-view image of the same tensile testing configuration illustrating the overall spool-grip setup during testing.

Testing the specimen in its original O-ring form retains key characteristics associated with industrial sealing components, including geometric constraints, manufacturing-induced variability, and potential internal defects. These features are often absent in standardized tensile specimens but can significantly influence mechanical performance in service conditions. As a result, the present approach provides a more representative assessment of load-bearing behavior relevant to sealing applications. The tensile testing configuration employed in this study is illustrated in Figure 3.

### 2.5. Data Processing and Analysis

Raw tensile testing data were acquired directly from the testing system in spreadsheet (Excel) format and consisted of time-resolved force and displacement measurements. Prior to analysis, the raw data were processed to remove non-physical artifacts such as preload effects, signal noise, and baseline offsets associated with the measurement system. Initial negative values recorded at the beginning of each test were excluded because they corresponded to system preload and piston positioning below the zero-force reference rather than the mechanical response of the elastomer. Data points recorded after the peak force, where force rapidly dropped due to specimen fracture, were also removed. Displacement values beyond specimen failure were excluded because they reflect piston motion rather than material response. The reported values correspond to measured displacement obtained from the change in spool separation during testing. Because the spool-based O-ring testing configuration does not produce a uniform strain field, conventional strain definitions are not directly applicable, and displacement-based metrics are used to provide a consistent and physically meaningful representation of the mechanical response across all test conditions.

Following data processing, each tensile response was truncated at the peak force corresponding to specimen rupture, and the peak force was used as the primary metric to quantify changes in tensile resistance. Additional mechanical parameters were derived directly from the force–displacement response to provide a more comprehensive description of material behavior. Because the spool-based configuration does not produce a well-defined stress field, conventional stress-based quantities are not reported, and the analysis is based on consistent force–displacement metrics across all test conditions.

#### 2.5.1. Energy Absorption

The mechanical energy absorbed during deformation was estimated as the area under the force–displacement curve up to the point of peak force. This quantity represents the work done on the specimen during loading and was calculated using numerical integration:

(1) $$E=\int_{0}^{\delta_{max}}F(\delta )\;d\delta$$

where $F\left(\delta \right)$ is the measured force and $\delta_{max}$ is the displacement at peak force. Numerical integration was performed using the trapezoidal rule. The resulting energy values are reported in units of lbf·in and provide a resistance to loading and ability to absorb mechanical work prior to rupture.

#### 2.5.2. Secant Stiffness

To characterize the stiffness of the elastomer under finite displacement, secant stiffness values were calculated at specified displacement levels. Secant stiffness was defined as:

(2) $$k_{secant}=\frac{F}{\delta }$$

where F is the force corresponding to a given displacement δ. In this study, stiffness values were evaluated at fixed fractions of the peak displacement (e.g., 25% and 50% of $\delta_{max}$) using interpolation. This approach provides a stable and reproducible measure of stiffness that is less sensitive to local fluctuations compared to differential (tangent) stiffness.

#### 2.5.3. Peak Displacement

The displacement corresponding to the maximum force was extracted for each test and used as an indicator of the material’s deformation capacity prior to failure. This parameter provides insight into changes in extensibility and ductility associated with hydrogen exposure.

#### 2.5.4. Late-Stage Instability (Custom Metric)

To quantify fluctuations in the force response near the end of loading, a late-stage instability parameter was defined as the standard deviation of the force signal over the final portion of the curve prior to peak force. Specifically, the last 15% of the data points before peak force were considered, and the instability metric was calculated as:

(3) $$\sigma_{late}=\mathrm{std}\;(F_{end})$$

where $F_{end}$ represents the force values within the selected final segment. This metric provides a quantitative measure of force variability near failure and serves as an indicator of potential instability in the deformation response. It should be noted that this is a descriptive parameter rather than a standardized mechanical property.

For each hydrogen aging condition, multiple replicate tests were performed. The tensile response was summarized using the arithmetic mean, while variability was quantified using the standard deviation. Results are reported as mean ± standard deviation, and shaded regions and error bars in the figures represent ±1 standard deviation of replicate measurements. No formal hypothesis testing (e.g., analysis of variance) was performed, as the primary objective was to identify pressure-dependent trends rather than statistical differences between individual conditions. The reported values provide a descriptive assessment of variability across replicate measurements.

To enable direct comparison of tensile response evolution across different hydrogen aging pressures and between materials, the force, displacement, and time histories were normalized with respect to their characteristic peak values. Specifically, force and displacement were normalized by their respective maximum values prior to rupture, while time was normalized by the time corresponding to the peak force. This normalization removes differences in absolute magnitude and test duration while preserving the shape of the loading and failure response, allowing pressure-dependent trends to be compared on a common dimensionless basis.

#### 2.5.5. Normalization Equations

(4) $$F_{norm}(t)=\frac{F(t)}{F_{max}}$$

(5) $$\delta_{norm}(t)=\frac{\delta (t)}{\delta_{max}}$$

(6) $$t_{norm}=\frac{t}{t_{max}}$$

where

$F\left(t\right)$ is the measured force,
$\delta \left(t\right)$ is the measured displacement,
$F_{max}$ and $\delta_{max}$ are the maximum force and displacement prior to rupture, and
$t_{max}$ is the time at which maximum force occurs.

Both raw and normalized responses were used in the analysis. Raw data were employed to quantify changes in tensile strength and energy absorption, while normalized data were used to visualize differences in displacement response, loading evolution, and time-dependent response independent of absolute strength levels. In graphical representations, the mean response curve represents the central tendency of the data, while shaded bands corresponding to one standard deviation indicate the dispersion among replicate specimens. This approach allows direct visualization of variability introduced by hydrogen aging while preserving the underlying trends in mechanical response.

All data processing, statistical analysis, and figure generation were performed using Python 3.12.7 (Python Software Foundation, Wilmington, DE, USA), employing the NumPy 1.26.4, Pandas 2.2.2, and Matplotlib 3.9.2 libraries, along with Pathlib for file handling, in combination with Microsoft Excel (Microsoft Corporation, Redmond, WA, USA).

#### 2.5.6. Statistical Definitions

For a set of n replicate measurements $x_{i}$:

(7) $$\overset{-}{x}=\frac{1}{n}\sum_{i=1}^{n}x_{i}$$

(8) $$\sigma =\sqrt{\frac{1}{n-1}\sum_{i=1}^{n}(x_{i}-\overset{-}{x}{)}^{2}}$$

where

$\overset{-}{x}$ is the mean value and
σ is the standard deviation.

### 2.6. Image Processing and Micro-Defect Quantification

Optical micrographs of FKM (Viton^®^) elastomer specimens were analyzed using a Python-based image processing workflow to quantify surface micro-defects [16,17]. All images were acquired at identical magnification (400×) under consistent illumination conditions. A spatial calibration factor (µm px^−1^), obtained from the 100 µm scale bar, was applied to convert pixel measurements to physical dimensions.

Images were converted to grayscale and contrast-enhanced using contrast-limited adaptive histogram equalization (CLAHE) [16]. Dark contrast features were highlighted using a morphology-based black-hat transformation. Binary segmentation was performed using Otsu thresholding, followed by morphological filtering to remove noise and improve feature continuity [16,17]. All processing parameters were kept constant to ensure consistency across samples.

Detected features were identified using connected-component analysis. For each feature, area and perimeter were measured, and an equivalent circular diameter was calculated. To exclude non-pit artifacts such as scratches or elongated features, shape filtering was applied using a circularity criterion:

(9) $$\mathrm{Circularity}=\frac{4\pi A}{P^{2}}$$

where A is the feature area and P is the perimeter. Only features with circularity ≥ 0.3 were retained.

The retained features were used to calculate micro-defect area fraction, defect density, and size distribution metrics. The analysis is based on optical contrast and does not directly resolve subsurface damage; therefore, detected features are interpreted as surface micro-defects for comparative evaluation across conditions.

## 3. Results

### 3.1. Mechanical Response Under Hydrogen Exposure

**Figure 4:**
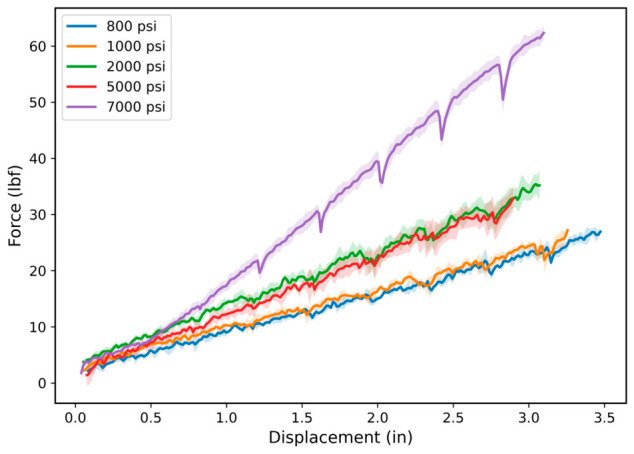
Force–displacement response of FKM after hydrogen exposure. Curves represent the mean force–displacement response of replicate FKM O-ring specimens tested after hydrogen exposure at each pressure condition (*n* ≥ 3 per condition), with shaded regions representing ±1 standard deviation. The results show increased load-bearing response at higher pressure, particularly at 7000 psi.

**Figure 5:**
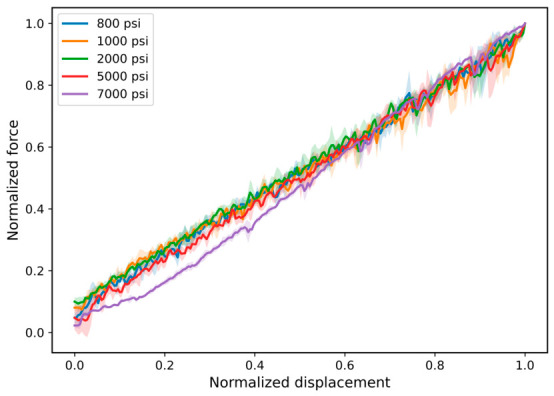
Normalized force–displacement curves for FKM specimens after hydrogen exposure at different pressures. The normalization is used to compare relative loading profiles independent of absolute force and displacement magnitude. Strain-stiffening behavior is evident at higher deformation levels, particularly for the 7000 psi condition. The normalized representation is intended for qualitative comparison only and does not constitute a constitutive analysis of elastomer mechanics.

Figure 4 shows the force–displacement response of FKM (Viton^®^) O-ring specimens after hydrogen exposure and is used to evaluate the effect of hydrogen pressure on tensile behavior. Figure 5 presents the corresponding normalized response. The results are shown using both raw and normalized representations, with shaded regions representing ±1 standard deviation across replicate tests. The tensile response exhibits a systematic increase in load-bearing capacity with increasing hydrogen pressure, with the 7000 psi condition showing the highest force response across the entire displacement range.

The force–displacement curves remain continuous without abrupt force drops, indicating the absence of brittle fracture or sudden structural failure. Minor fluctuations in force are observed, particularly at higher pressure conditions such as 7000 psi; these are attributed to localized deformation instabilities and may arise from microstructural damage evolution, local stress redistribution, or contact effects between the specimen and the spool fixtures. Such fluctuations are typical of elastomeric materials under large deformation and do not disrupt the overall load-bearing response.

An increase in stiffness at higher displacement levels is observed, particularly for the 7000 psi condition, consistent with strain-stiffening behavior commonly exhibited by elastomers. This suggests that FKM maintains structural integrity under hydrogen exposure. The increased force response at higher pressures may be associated with pressure-induced constraint effects, including reduced free volume and restricted chain mobility within the polymer network. However, this interpretation is based on observed mechanical trends and is presented as a plausible explanation rather than a definitive mechanism. Direct validation would require complementary characterization techniques such as dynamic mechanical analysis (DMA), gas sorption measurements, or free-volume analysis.

While the force–displacement response provides insight into load-bearing capacity, it does not distinguish whether the observed changes arise from differences in magnitude or from modifications in displacement response.

Figure 5 presents the normalized force–displacement response used to compare the relative shape of the mechanical behavior independent of absolute force and displacement magnitude. The normalized curves show broadly similar loading profiles across the investigated pressure conditions, suggesting that the relative progression of loading remains comparable after hydrogen exposure. However, this normalization is used only for comparative visualization and does not establish preservation of the deformation or failure mechanism. Mechanistic interpretation would require additional strain-based analysis, constitutive modeling, or complementary viscoelastic characterization.

Therefore, Figure 5 should be interpreted as indicating similarity in normalized response profiles, while quantitative evaluation of pressure-dependent behavior is based on the measured force–displacement metrics, including peak force, energy absorption, secant stiffness, and displacement at peak force.

**Figure 6:**
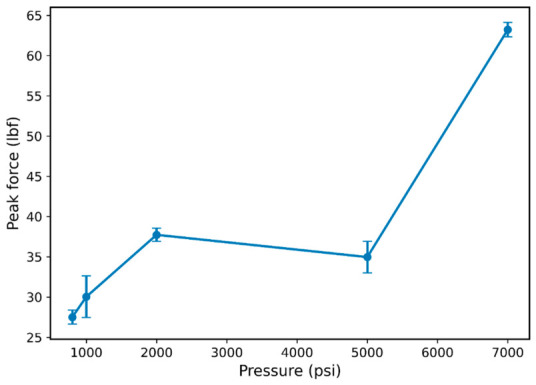
Peak force as a function of hydrogen pressure. Values represent mean ± standard deviation based on replicate O-ring specimens at each pressure condition (*n* ≥ 3 per condition), showing a non-monotonic trend with a pronounced increase at high pressure.

To further quantify the influence of hydrogen pressure on mechanical performance, Figure 6 shows the variation in peak force with hydrogen pressure, providing a quantitative measure of tensile strength evolution. The mean peak force increased from approximately 27.5 lbf at 800 psi to 30.0 lbf at 1000 psi (≈9% increase) and further to 37.8 lbf at 2000 psi (≈37% increase relative to 800 psi). However, at 5000 psi, the peak force decreased slightly to approximately 35.0 lbf (≈7% reduction relative to 2000 psi), indicating a non-monotonic response. At 7000 psi, a pronounced increase in peak force was observed, reaching approximately 63.5 lbf, corresponding to an increase of about 131% relative to the 800 psi condition and approximately 81% relative to 5000 psi.

This non-monotonic trend, characterized by an intermediate reduction followed by a substantial increase at higher pressure, indicates that the behavior of FKM is governed by competing mechanisms rather than a simple monotonic trend. The pronounced increase at 7000 psi suggests a dominant stiffening or constraint-driven response at elevated hydrogen pressures.

However, peak force alone does not fully capture the material response, as it does not account for the deformation history prior to failure. These interpretations are based on observed mechanical trends and optical analysis; direct validation would require viscoelastic and transport measurements such as dynamic mechanical analysis (DMA) or hydrogen sorption analysis. Therefore, energy absorption was evaluated to provide a more comprehensive measure of mechanical performance.

**Figure 7:**
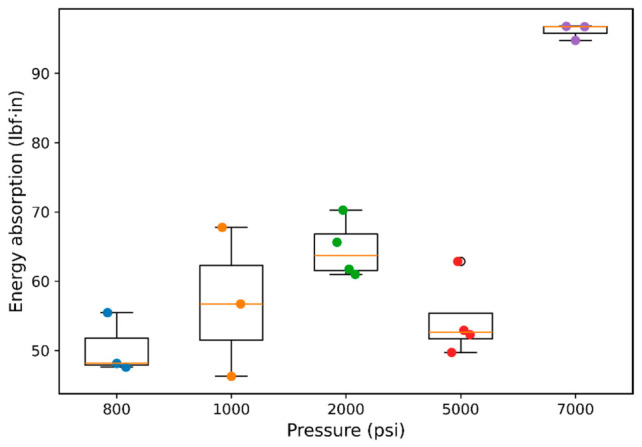
Energy absorption behavior of FKM elastomer. Energy absorbed during deformation (area under the force–displacement curve up to peak force) as a function of hydrogen pressure, exhibiting a non-monotonic trend with a significant increase at high pressure. Different colors correspond to different hydrogen pressure conditions.

Because peak force alone does not capture the full deformation history, Figure 7 shows the energy absorption behavior of FKM as a function of hydrogen pressure. The energy absorption increased from approximately 48.0 lbf·in at 800 psi to about 56.7 lbf·in at 1000 psi (≈18% increase) and further to approximately 63.8 lbf·in at 2000 psi (≈33% increase relative to 800 psi). However, at 5000 psi, the energy absorption decreased to approximately 52.4 lbf·in, indicating a non-monotonic trend consistent with the peak force behavior. At 7000 psi, a substantial increase in energy absorption was observed, reaching approximately 96.5 lbf·in, corresponding to nearly a 100% increase relative to the baseline condition. This pronounced increase indicates that the material exhibits significantly enhanced resistance to deformation and failure at elevated pressure.

The observed non-monotonic trend suggests the presence of competing mechanisms at intermediate pressures, while the strong increase at 7000 psi indicates a transition toward a more constrained and load-bearing network structure under high-pressure hydrogen exposure. The non-monotonic variation in energy absorption further indicates that the tensile response alone is insufficient to fully resolve the underlying deformation mechanisms, suggesting that additional time-dependent viscoelastic characterization is necessary. While energy absorption reflects the combined effects of strength and deformation, it does not explicitly describe the deformation capacity of the material. To further understand changes in extensibility, the peak displacement behavior was evaluated.

**Figure 8:**
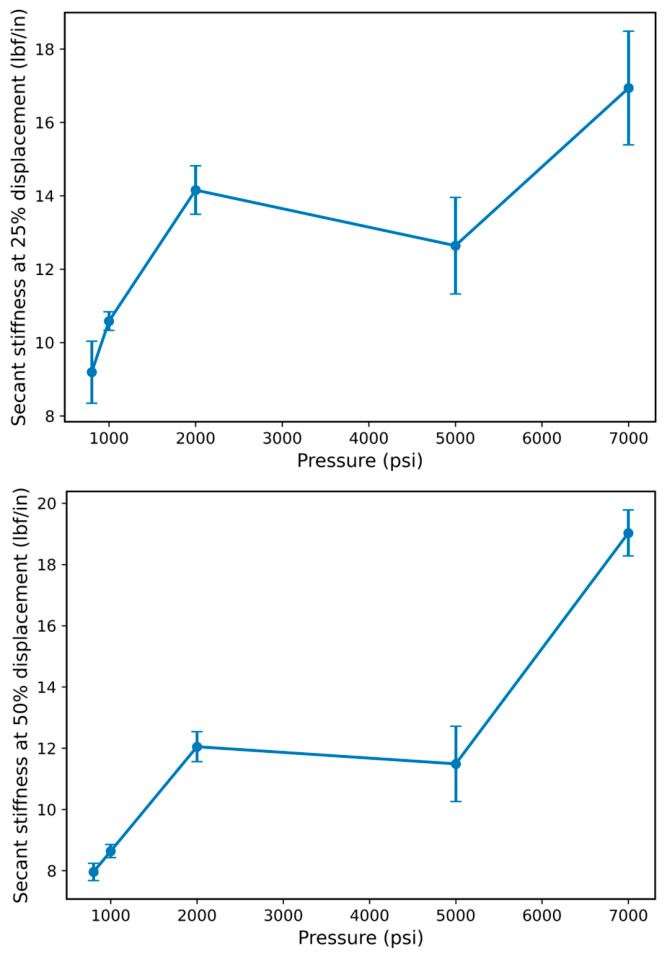
Secant stiffness variation with hydrogen pressure. Secant stiffness values evaluated at 25% and 50% of peak displacement are reported as mean ± standard deviation based on replicate O-ring specimens at each pressure condition (*n* ≥ 3 per condition).

To further understand resistance to deformation across different strain levels, Figure 8 presents the secant stiffness variation with hydrogen pressure, where the secant stiffness is evaluated at 25% and 50% of peak displacement. Secant stiffness values are reported in units of lbf/in based on the force–displacement response. Both stiffness measures exhibit a similar non-monotonic trend with increasing pressure, indicating consistent mechanical behavior across different deformation levels. At lower pressures (800–1000 psi), a moderate increase in stiffness is observed relative to baseline, suggesting an initial increase in resistance to deformation. The stiffness reaches a local maximum at approximately 2000 psi, indicating enhanced load-bearing capability under intermediate pressure conditions. Beyond this point, a reduction in stiffness is observed at 5000 psi, followed by a pronounced increase at 7000 psi.

Importantly, the trends obtained at 25% and 50% of peak displacement remain consistent, indicating that the pressure-dependent mechanical response is not limited to a specific stage of loading but persists across both early-stage and intermediate response regimes. This agreement suggests that the observed changes in stiffness reflect intrinsic modifications to the material response rather than localized or strain-specific effects. The increase in stiffness at higher pressures is indicative of increased resistance to deformation, which may be associated with pressure-induced constraint effects and reduced chain mobility. Conversely, the reduction in stiffness observed at intermediate pressures suggests the presence of competing mechanisms that temporarily reduce the material’s resistance to deformation.

Overall, the non-monotonic stiffness response reinforces the trends observed in peak force and energy absorption, indicating that hydrogen exposure influences the mechanical behavior of FKM through a complex interplay of mechanisms rather than a simple monotonic degradation or softening process.

**Figure 9:**
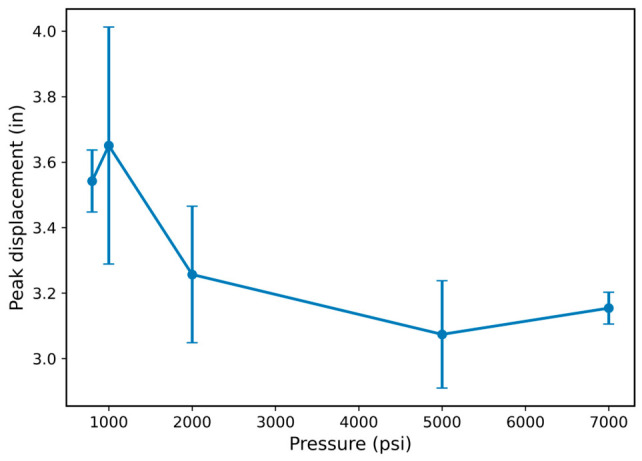
Peak displacement as a function of hydrogen pressure. Values represent mean ± standard deviation based on replicate O-ring specimens at each pressure condition (*n* ≥ 3 per condition), showing reduced displacement at higher pressures and indicating decreased extensibility of the elastomer.

In contrast to strength-related metrics, Figure 9 shows the variation in peak displacement with hydrogen pressure. The peak displacement increased slightly from approximately 3.54 in at 800 psi to about 3.66 in at 1000 psi (≈3% increase), indicating minimal influence of hydrogen at low pressure. However, a clear reduction in peak displacement was observed at higher pressures, decreasing to approximately 3.27 in at 2000 psi (≈8% decrease), 3.07 in at 5000 psi (≈13% decrease), and about 3.15 in at 7000 psi (≈11% decrease relative to 800 psi).

This reduction in displacement at elevated pressures indicates a decrease in material extensibility, suggesting that the polymer network becomes increasingly constrained under high-pressure hydrogen exposure. When considered together with the simultaneous increase in peak force and energy absorption at high pressure, the results indicate a transition toward a stiffer and more load-bearing network that resists displacement but reaches failure at lower displacement levels. The reduction in peak displacement at elevated pressures, combined with the non-monotonic variation in strength and energy absorption, suggests that the mechanical response is governed by competing mechanisms that cannot be fully resolved through monotonic tensile testing alone, highlighting the need for time-dependent viscoelastic characterization such as DMA.

Taken together, these results indicate that hydrogen exposure leads to a transition toward a stiffer and more constrained material response at high pressure, governed by competing mechanisms that cannot be fully resolved using monotonic tensile testing alone. This behavior motivates the need for time-dependent viscoelastic characterization in future work.

### 3.2. Quantitative Defect Analysis

While tensile testing provides insight into the global mechanical response, it does not directly capture the microstructural changes associated with hydrogen exposure. To address this, representative optical micrographs, corresponding binary masks, and quantitative metrics derived from image segmentation were used to evaluate pressure-dependent surface damage.

Table 3 summarizes the quantitative image analysis results, showing an increase in micro-defect accumulation across the analyzed pressure conditions. Image analysis was performed for selected pressures (800, 1000, and 7000 psi) to provide representative comparison across low, intermediate, and high-pressure exposure levels. Although mechanical testing covered a broader pressure range, the absence of image-based data at intermediate pressures (2000 and 5000 psi) limits direct correlation between surface defect evolution and mechanical response across all conditions.

The variability in micro-defect metrics, particularly at higher pressures, is relatively large and reflects the heterogeneous and localized nature of surface damage in elastomeric materials. Defect formation occurs in discrete regions influenced by local microstructural features, leading to significant variation across individual fields of view. Accordingly, the quantitative results are interpreted as a comparative assessment across the analyzed pressure conditions rather than a strictly predictive trend, and the observed variability highlights the limitations of optical surface analysis in fully capturing damage evolution.

The mean micro-defect area fraction increased from approximately 0.038% at 800 psi to 0.085% at 1000 psi and 0.139% at 7000 psi, corresponding to about a 2.24-fold increase at 1000 psi and a 3.64-fold increase at 7000 psi relative to the 800 psi condition (Table 3). A similar trend was observed in micro-defect density, which increased from approximately 57.5 defects/mm^2^ at 800 psi to 98.6 defects/mm^2^ at 1000 psi and 130.9 defects/mm^2^ at 7000 psi. The increase in area fraction is consistent with the rise in defect density, indicating that surface degradation is primarily driven by the accumulation of multiple micro-scale defects rather than isolated growth events.

Despite this overall trend, the distribution of micro-defects was not uniform across all image fields. Variability increased substantially with pressure, as reflected by the coefficient of variation in micro-defect area fraction, which rose from approximately 38% at 800 psi to about 92% at 1000 psi and 125% at 7000 psi. This increase reflects the inherently heterogeneous and localized nature of surface damage in elastomeric materials, where defect formation occurs in discrete regions, leading to significant spatial variation across image fields. Consequently, the observed statistical dispersion is influenced by both the stochastic nature of defect formation and the limited number of analyzed fields of view. The quantitative results are therefore interpreted as indicative of general trends rather than precise deterministic relationships. Because the standard deviation is relatively large compared to the mean values, the graphical representation of mean ± standard deviation may extend toward non-physical ranges; however, the underlying measured defect metrics are strictly non-negative.

The increasing scatter suggests that damage becomes more spatially heterogeneous at higher pressures, with localized regions exhibiting elevated defect concentration. In particular, individual fields at 1000 psi and 7000 psi showed markedly higher area fraction and defect density, indicating the presence of localized damage regions.

The pressure-dependent behavior suggests that surface damage evolution may involve both enlargement of pre-existing weak regions and formation of new localized defects during hydrogen exposure, decompression, and subsequent mechanical loading. Under high-pressure exposure, hydrogen may diffuse into the elastomer network and accumulate near microstructural heterogeneities such as filler–matrix interfaces or regions of reduced constraint. During depressurization, the resulting pressure gradients may generate localized stresses that contribute to surface disruption and defect formation. Accordingly, the observed features are interpreted as post-exposure surface micro-defects influenced by coupled hydrogen sorption, decompression-induced effects, and mechanical loading, rather than direct evidence of a single damage mechanism.

Texture-based metrics provide additional supporting information. Surface roughness did not exhibit a consistent monotonic trend with pressure, indicating that it is not a reliable standalone indicator of hydrogen-induced damage in this dataset, even though image-based defect analysis indicates pressure-dependent surface changes.

The present analysis did not identify consistent blister-like features across the investigated pressure range. Accordingly, the observed changes are more appropriately interpreted as pressure-associated micro-defect accumulation and increasing surface heterogeneity rather than definitive evidence of macroscopic blister formation.

Overall, the microscopy results demonstrate a pressure-dependent increase in surface micro-damage in FKM elastomers, accompanied by a transition toward more heterogeneous and spatially localized degradation patterns. These observations provide independent microstructural evidence supporting the mechanical results and are consistent with the increased stiffness and load-bearing response observed at higher pressures. A quantitative correlation between defect density and mechanical response remains to be established. The quantitative trends summarized in Table 3 provide a consolidated view of pressure-dependent surface degradation across the analyzed pressure conditions.

### 3.3. Qualitative Microstructural Observations

**Figure 10:**
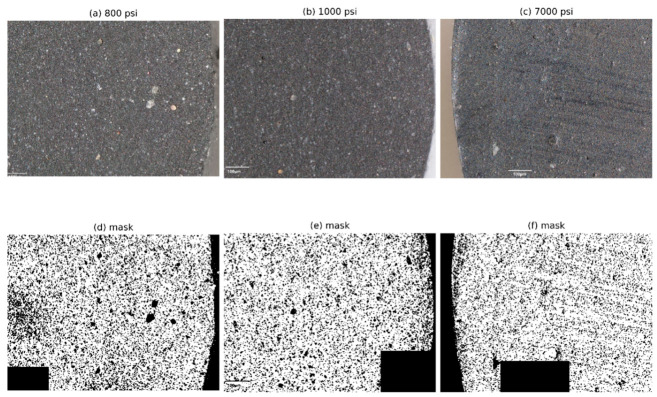
Representative optical micrographs and corresponding binary masks of FKM elastomer after hydrogen exposure. (**a**–**c**) Optical micrographs of FKM surfaces exposed to hydrogen at 800 psi, 1000 psi, and 7000 psi, respectively. (**d**–**f**) Corresponding binarized images used for quantitative defect identification. Dark regions represent segmented micro-defects. An increase in defect density and the emergence of elongated and clustered features are observed with increasing pressure. The observed dark contrast features are identified based on optical image segmentation and do not provide direct evidence of void formation or subsurface damage. Accordingly, these features are interpreted as surface defect indicators for comparative analysis across conditions. All optical micrographs were acquired at 300× magnification with a scale bar of 100 µm.

Figure 10 presents representative optical micrographs and corresponding binary masks used to visualize the spatial evolution of surface damage with increasing hydrogen pressure. The binarized images at 7000 psi reveal a substantial increase in defect density compared to lower-pressure conditions, along with a more widespread distribution of dark regions across the surface. In addition to increased density, elongated and directionally aligned features are observed, suggesting anisotropic damage evolution or preferential pathways within the material. Localized regions of high defect concentration are also evident, indicating non-uniform damage accumulation potentially influenced by stress or diffusion gradients. Furthermore, localized coalescence of smaller defects into larger irregular features is occasionally observed, reflecting a transition from isolated surface defect features to more interconnected damage structures at elevated pressure.

The identification of surface features in this study is based on optical contrast and image segmentation and does not provide direct confirmation of void formation or subsurface structural damage. The observed dark contrast regions may arise from a combination of surface roughness, microstructural disruption, illumination effects, or imaging artifacts. Accordingly, the results are interpreted as relative indicators of surface degradation rather than definitive evidence of specific defect morphology.

**Figure 11:**
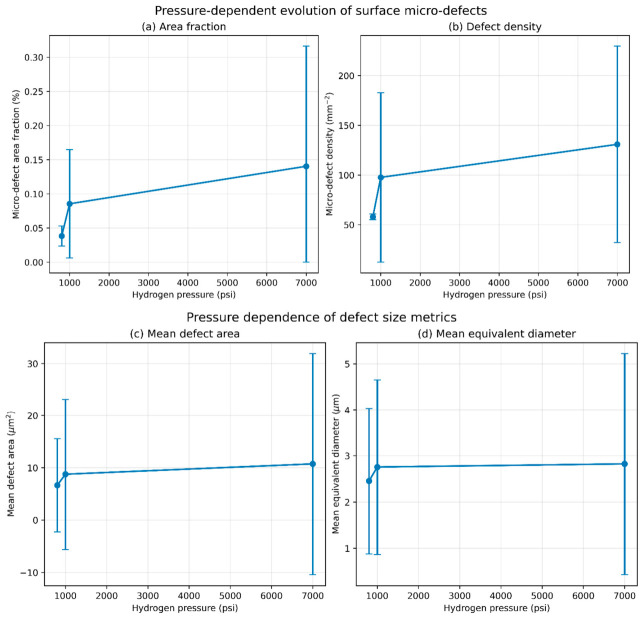
Quantitative analysis of pressure-dependent micro-defect evolution in FKM elastomer. (**a**) Micro-defect area fraction, (**b**) defect density, (**c**) mean defect size, and (**d**) characteristic defect size (P90 diameter) as a function of hydrogen exposure pressure. Values represent mean ± standard deviation based on analyzed optical micrographs/fields of view at each selected pressure condition. The number of analyzed fields of view is reported in Table 3. The relatively large variability reflects the heterogeneous and localized nature of defect formation in elastomeric materials under hydrogen exposure, and the error bars represent statistical dispersion across analyzed fields of view.

Figure 11 presents the quantitative variation in micro-defect characteristics as a function of hydrogen pressure. The mean defect size exhibited a moderate increase with hydrogen pressure. The average defect area increased from 6.64 µm^2^ at 800 psi to 10.73 µm^2^ at 7000 psi (≈60% increase), while the mean equivalent diameter increased from 2.45 µm to 2.82 µm over the same range. However, this increase in defect size is smaller than the corresponding increase in defect density and area fraction, suggesting that damage evolution is primarily associated with the formation of new micro-defects rather than extensive growth of existing ones. The large standard deviation at higher pressures further indicates the presence of localized larger features, potentially associated with coalescence or displacement-assisted processes.

Because the specimens were imaged after hydrogen exposure, depressurization, and tensile testing, the observed surface features may reflect combined effects of hydrogen-induced damage and mechanically assisted opening or growth of pre-existing defects. Based on optical surface observations alone, it is not possible to distinguish whether the features originate from enlargement of pre-existing regions, newly formed defects during exposure or decompression, or mechanically induced damage during testing.

## 4. Conclusions

In this study, the mechanical response and surface degradation behavior of FKM elastomer under high-pressure hydrogen exposure were systematically investigated using tensile testing and quantitative optical microscopy analysis. The results demonstrate that hydrogen exposure induces significant changes in both mechanical performance and surface morphology. The tensile response exhibited a non-monotonic dependence on hydrogen pressure, with a pronounced increase in peak force, stiffness, and energy absorption at 7000 psi. At the same time, the displacement capacity decreased, indicating a transition toward a stiffer and more constrained material response under high-pressure conditions. Quantitative image analysis revealed a clear increase in micro-defect area fraction and defect density with increasing pressure, confirming progressive surface damage accumulation. In contrast, the mean defect size showed only a moderate increase, suggesting that hydrogen exposure primarily promotes the nucleation of new micro-defects rather than substantial growth of existing ones. The large variability observed at higher pressure further indicates the development of spatially heterogeneous damage, including localized regions of intensified degradation. Qualitative microstructural observations supported these findings, showing a higher density of defects and the emergence of elongated and clustered features at elevated pressures, particularly at 7000 psi. These features are consistent with localized damage accumulation and possible displacement-assisted mechanisms. Overall, the combined mechanical and microscopy results suggest that hydrogen-induced degradation in FKM elastomer is associated with increased formation of micro-defects relative to their growth, accompanied by increasing spatial heterogeneity at higher pressures. These findings highlight the importance of integrating microstructural characterization with mechanical testing to better understand material performance under hydrogen service conditions.

Future work should focus on incorporating additional characterization techniques to better understand the underlying damage mechanisms. These may include scanning electron microscopy (SEM), atomic force microscopy (AFM), micro-computed tomography (micro-CT), cross-sectional analysis, dynamic mechanical analysis (DMA), and hydrogen sorption measurements, which are essential for validating the proposed interpretations.

## Figures and Tables

**Table 1:** Physical and specification parameters of the FKM (Viton^®^) O-ring specimens used in this study. The O-rings correspond to AS568 Dash-214 geometry and represent commercially available sealing materials with standardized dimensions and properties relevant to industrial applications.

Property	FKM (Viton^®^) O-Ring
Material type	Fluorocarbon elastomer (FKM)
Standard	SAE AS568 (geometry specification)
Hardness	60 Shore A (soft)
Inner diameter	0.984 in (25.0 mm)
Outer diameter	1.262 in (32.05 mm)
Cross-section	0.139 in (3.53 mm)

**Table 2:** Summary of specimen characteristics, hydrogen aging conditions, and experimental parameters used in this study. All conditions were maintained constant except for hydrogen pressure to enable direct evaluation of pressure-dependent mechanical behavior.

Parameter	Value
Material	FKM (Viton^®^) O-rings (see Table 1)
Specimen geometry	O-rings
Cross-section diameter	Identical for all tests
Hydrogen aging pressure	800–7000 psi
Aging duration	192 h
Aging temperature	Room temperature
Hydrogen purity	High-purity H_2_
Number of replicates	*n* ≥ 3 per condition

**Table 3:** Summary of surface micro-defect characteristics of FKM elastomer after hydrogen exposure at different pressures. Values represent mean ± standard deviation based on multiple analyzed image fields (n), where n denotes the number of independent micrographs (fields of view) used for quantitative defect analysis at each pressure condition.

Pressure (psi)	n	Micro-Defect Area (%)	Micro-Defect Density (mm^−2^)	P90 Diameter (µm)	Small Defects (%)	Medium Defects (%)	Top-3 Defect Area (%)	Fold Increase (Area)
800	3	0.038 ± 0.015	57.5 ± 22.0	3.86 ± 0.48	50.5 ± 9.3	49.5 ± 9.3	59.2 ± 7.5	1
1000	4	0.085 ± 0.078	98.6 ± 79.3	4.32 ± 0.87	48.2 ± 8.8	51.8 ± 8.8	56.1 ± 10.2	2.24
7000	3	0.139 ± 0.174	130.9 ± 95.5	5.23 ± 1.21	51.7 ± 11.4	48.3 ± 11.4	42.0 ± 12.6	3.64

## Data Availability

Data are contained within the article. Further inquiries can be directed to the corresponding author.
